# A study of the correlation between obesity and intestinal flora in school-age children

**DOI:** 10.1038/s41598-018-32730-6

**Published:** 2018-09-28

**Authors:** Xiaolin Gao, Ruizhen Jia, Liang Xie, Linghan Kuang, Ling Feng, Chaomin Wan

**Affiliations:** 1 Department of Paediatrics, West China University Second Hospital, Sichuan University, Chengdu, Sichuan 610041 China; 2 Open Laboratory, West China Institute for Women’s and Children’s Health, Chengdu, Sichuan 610041 China; 3 Pulmonary Vascular Remodelling Research Unit Laboratory, West China Institute for Women’s and Children’s Health, Chengdu, Sichuan 610041 China; 4 Department of Laboratory Medicine, Sichuan University West China Second Hospita, Chengdu, Sichuan 610041 China; 5 Key Laboratory of Birth Defects and Related Diseases of Women and Children, Sichuan University, Ministry of Education, Chengdu, Sichuan China

## Abstract

With the improvement of living standards and dietary changes, childhood obesity has increased worldwide. This study aimed to understand the differences of intestinal flora structure between obese and normal children at school-age. Using the next generation sequencing platform, Illumina Miseq, 16S rDNA high-throughput sequencing technology, we analyzed the diversity and relative abundance of intestinal flora in 39 obese and 38 normal control school-age children. First, we categorized gut bacteria on the basis of their Operational taxonomic units (OTUs) using the RDP 16s rRNA database in RDP classifier. The alpha (α) diversity was used to measure the diversity within a sample and is calculated as a value for each sample. The beta (β) diversity was used to compare different samples and to measure the dissimilarity between each other sample. Our results indicated that intestinal flora in obese children showed lower diversity than normal controls. Significant differences of relative abundance of intestinal flora were detected at multiple levels of classifications. Identification of intestinal flora with significant difference between obese and normal children may provide important information to uncover the roles of these specific bacteria in the development of obesity and find new strategy to prevent and treat obesity through intervening the intestinal flora.

## Introduction

With the improvement of living standards and dietary changes, childhood obesity has become increasingly identified worldwide, and children have been found to be affected by obesity at younger ages^1,2^. According to the World Health Organization (WHO) report, the rate of global obesity has exhibited a rapid upward trend, leading to an obese population that is double the size of that identified in 1980. Currently, it has been estimated that there are more than 40 million obese children worldwide^3^. Among the non-genetic factors associated with obesity, the effects of the intestinal flora have been recognized another category of obesity regulators, as the correlation between intestinal flora changes and body weight has been detected in a number of studies for animal obesity models^4–6^. However, the status of intestinal microbiome in children with obesity have not been well studied, especially regarding children at school-age. Consistent with previous studies^7,8^, using real-time quantitative PCR (qPCR), we demonstrated that obesity was associated with changes in intestinal *Bifidobacteria* (B) and *Escherichia*. *coli* (C), and a positive correlation was observed between the *B/E* ratio among school-age children^9^.

While the real-time PCR with fluorescent-labeled primer is widely used to quantify the nucleotides with many advantages, it is very costly as well as labor- and time-consuming, because it is necessary to design specific primer pairs and establish standard curve for each specific bacteria flora^10,11^. In addition, a study with real-time PCR will have to focus on certain species with limited number of species to examine. Other traditional microbiological identification methods, such as bacterial culture, and denaturing gradient gel electrophoresis (DGGE), also have the similar limitations in the study of complex intestinal flora. Fortunately, the high-throughput sequencing techniques become available and it allows us to sequence millions of DNA molecules at the same time and provide a data pool to cover the entire microbiome in the gut. Because this sequencing method preserve the integrity of whole microbiome and calculate the amount of different species according to the number of matched sequences to a specific species, the measurement of relative abundance of the species at multiple levels using a more sophisticated algorithm^11^. The rDNA gene encoding 16S rRNA is the most commonly used target for studying bacterial evolution and classification. Without a requirement of cloning and screening, this new high-throughput technology is capable of generating large amounts of data within a short period of time, and can provide information regarding the composition, distribution and relative abundance of species in microbial communities. In our study, we used the Illumina Miseq. 16S rDNA high-throughput sequencing technology to study the differences of the intestinal microbial flora in school-age children affected by obesity along with normal counterparts.

## Results

### Basic participant information

A total of 77 subjects were included in the obesity group (n = 39) and control groups (n = 38) (Table 1). No significant differences in age, gender or height (*P* > 0.05) were detected between the two groups. Body weight and body mass index (BMI) were significantly different between the obesity group and the control group (*P* = 0.001).

**Table 1:** Characteristics of the participants in this study ($\overline{x}$ ± *s*).

	Obesity (*n* = 39)	Control (*n* = 38)	P-value
Male	20	20	0.231
Female	19	18	0.181
Age	6.8 ± 1.6	6.0 ± 2.7	0.065
Height (m)	1.23 ± 0.14	1.18 ± 0.21	0.012
Weight (kg)	35.4 ± 5.8	21.0 ± 2.3	0.000
BMI(kg/m^2^)*	25.2 ± 3.1	16.2 ± 2.6	0.001

^*^BMI (Body Mass Index) = weight/height^2^ (kg/m^2^).

### Basic sequencing information

We have deposited the bacterial sequencing read data to NCBI(Codes No: SRA759603).

After executing sample assembly and quality control using the Illumina Miseq platform, 150026 effective sequences of DNA encoding 6S rRNA were obtained. The maximum of read length was 25364 bps and the minimum of read length was 8707 bps. An average of read length of 19982 ± 2269.60 bps was obtained from obesity group, and an average of read length of 18973 ± 4175.33 bps was obtained from control group (*P* = 0.000). With a 97% similarity as the cutoff value, 8409 classification units were identified, of which an average of 99.10 ± 3.40 were derived from obesity group and an average of 109.58 ± 4.80 were derived from control group (*P* < 0.05).

Using the designated software provided by sequencing provider (Huada Gene Technology Co., Ltd), data meeting the quality criteria for sequencing depth, coverage and uniformity were included for further analysis of species abundance and and diversity.

The classification of gut bacteria was based on their Operational taxonomic units (OTUs) using the RDP 16s rRNA database in RDP classifier (v2.2; https://rdp.cme.msu.edu/classifier/classifier.jsp)

### Alpha (α) diversity analysis

**Figure 1:**
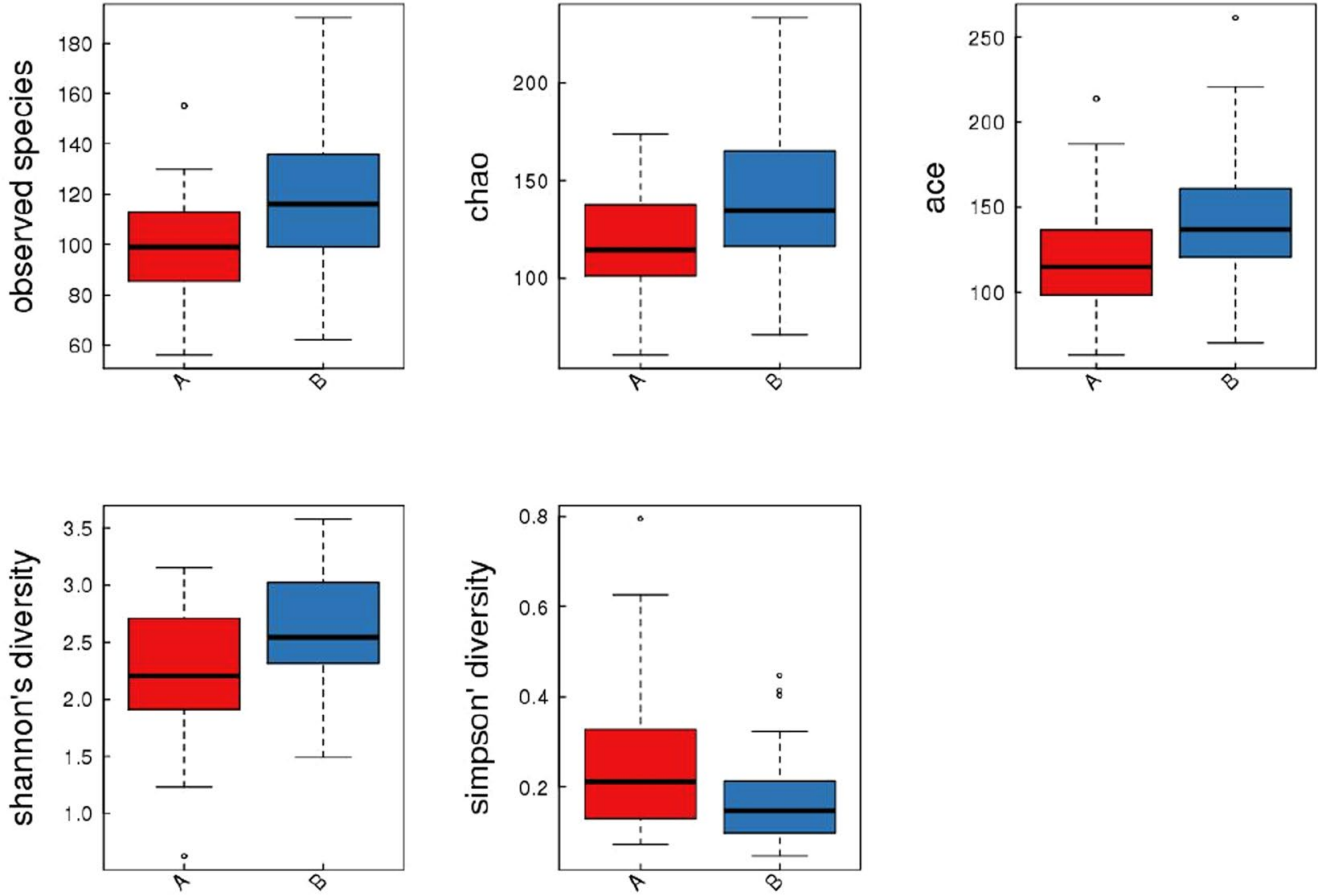
Box-plot of α diversity of the obesity group and the control group. Red boxes represent the obesity group, and blue boxes represent the control group.

The α diversity is used to measure the diversity within a sample and is calculated as a value for each sample. Four metrics were used for α diversity analysis: the Observed species, Chao, ACE index, Shannon index and Simpson index. These indexes measure the richness and evenness; the higher values of the first four indexes represent the higher richness. In contrast, the lower value of Simpson index represents higher richness. To ensure the numbers of sequences were sufficient to calculate the alpha diversity, rarefaction plot was established for each index. An example of rarefaction plot is shown in Supplementary Fig. 1. The values of these indexes in obesity and control groups were plotted and compared (Fig. 1), all of these indexes showed statistical significance (*P* = *0*.*002*, *0*.*008*, *0*.*004*, *0*.*003*, *0*.*020* for Observed species, Chao, ACE, Shannon and Simpson, respectively).

### Beta (β) diversity analysis

**Figure 2:**
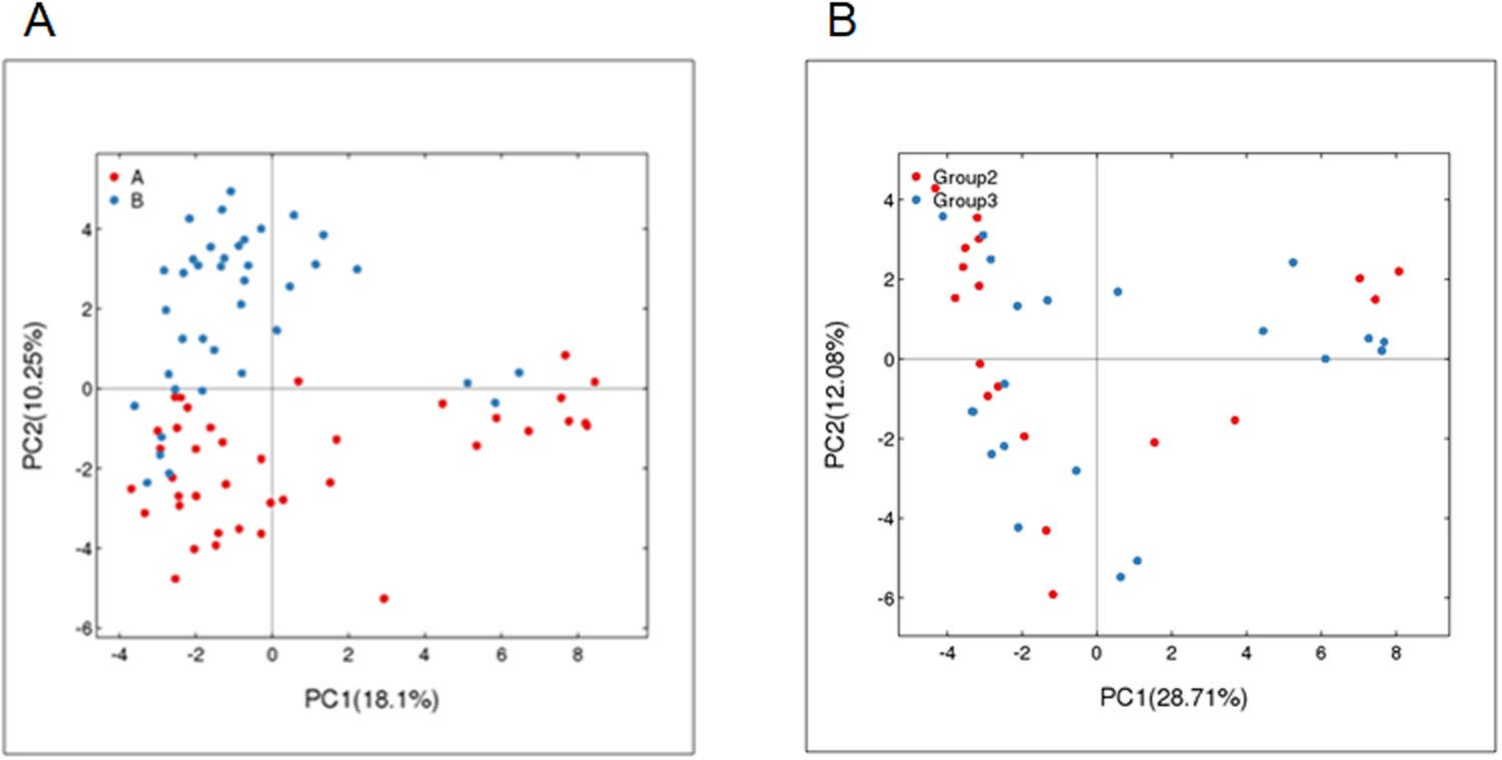
(**A**) PCA analysis of the obesity group and the control group based on OTU abundance. Red points indicate the obesity group, and blue points represent the control group. (**B**) PCA analysis based on OTU abundance in the obese group the obesity group data grouped by a BMI threshold of 25. Group 1 (blue) has BMI values ≥ 25, Group 2 (red) has BMI values < 25.

Beta diversity is used to compare different samples and it provides a measurement of distance or dissimilarity between each other sample. The distributions of the examined 77 samples of obesity and control groups are plotted in Fig. 2A. PC1 is the first principle coordinate, accounting for 18.1% of the total flora diversity. PC2 is the vertical axis, which accounts for 10.25% of the total flora diversity. While most of the blue points (control) distribute in the upper left quadrant of the figure, most of the red points (obesity) distribute in the bottom quadrants of the graph. It is suggested that there are a distinct clustering pattern between the control and obesity groups. Of note, four blue points (C16, C23, zheng25, zheng26) in the control group might be outliers, which were not completely separated from the points of the obesity group. Next, we further analyzed the obesity group by separating them into two groups using BMI 25. Group 1 (blue) has BMI values ≥ 25, Group 2 (red) has BMI values < 25. There was no distinct separation was observed in these two sub-groups of obesity groups: the blue and red dots evenly distributed. These data suggest that the samples from the same group (obesity or control groups) share the similarity but the samples from the different groups show the overall dissimilarity.

### Relative abundance analysis

Comparisons of the relative abundance of gut bacteria were performed at the levels of the phylum and species.

A total of 13 phyla of bacteria were detected, and differences were identified between the two groups. Among the 13 phyla of bacteria detected, five of them were significantly different between the obesity group and the control group (FDR ≤ 0.05, and/or *P* < 0.05), as detailed in the Table 2. Specifically, *Bacteroidetes* was the predominant phylum in both studied groups, but it was significantly higher in the obesity group than in the control group (the relative median abundance were 63.607% and 50.072%, respectively). In contrast, the relative abundances of *Candidatus*, *Actinobacteria*, *Firmicutes*, and *Verrucomicrobia* were higher in the control group than in the obesity group. There were no significant differences in the remaining phyla between the obesity group and the control group.

**Table 2:** Comparisons of relative abundance of gut bacteria between the obesity and control groups at the level of Phylum.

Phylum	Obesity (Q1, Q3)	Obesity IQR	Control (Q1, Q3)	Control IQR	P-value	FDR
**Phylum show higher relative abundance in control group than in obesity group**						
Candidatus_Saccharibacteria	0.0037 (0 × 10^−4^ to 0.005 × 10^−3^)	0.005	**0.072** (0.006 × 10^−4^ to 0.241 × 10^−3^)	0.235	0.000	**0**.**000**
Actinobacteria	0.138 (0.055 × 10^−4^ to 0.421 × 10^−3^)	0.366	**0.841** (0.162 × 10^−4^ to 2.491 × 10^−3^)	2.329	0.000	**0**.**000**
Firmicutes	29.236 (21.179 × 10^−4^ to 40.813 × 10^−3^)	19.633	**44**.**275** (30.884 × 10^−4^ to 67.032 × 10^−3^)	36.148	0.001	**0**.**003**
Verrucomicrobia	0 (0 × 10^−4^ to 0 × 10^−3^)	0.000	0 (0 × 10^−4^ to **0.04** × **10**^−3^)	0.040	0.006	**0**.**016**
**Phylum shows higher relative abundance in obesity group than in control group**						
Bacteroidetes	**63**.**607** (55.231, 75.166)	19.935	50.072 (24.965, 63.261)	38.296	0.000	**0**.**000**
**Phylum show no difference in relative abundance between obesity and control groups**						
Proteobacteria	2.313 (1.183, 5.314)	4.131	1.362 (0.227, 3.295)	3.068	0.051	0.119
Unclassified	0.0479 (0.025, 0.092)	0.067	0.0710 (0.023, 0.418)	0.395	0.061	0.122
Armatimonadetes	0 (0, 0)	0.000	0 (0, 0)	0.000	0.328	0.447
Deinococcus_Thermus	0 (0, 0)	0.000	0 (0, 0)	0.000	0.328	0.447
Fusobacteria	0 (0, 0.012)	0.012	0 (0, 0.004)	0.004	0.351	0.447
Tenericutes	0 (0, 0)	0.000	0 (0, 0)	0.000	0.328	0.447
Lentisphaerae	0 (0, 0)	0.000	0 (0, 0)	0.000	0.612	0.659
Synergistetes	0 (0, 0)	0.000	0 (0, 0)	0.000	0.587	0.659
Cyanobacteria_Chloroplast	0 (0, 0)	0.000	0 (0, 0)	0.000	0.706	0.706

Data are present as median (Q1, Q3) and interquartile range (IQR = Q3–Q1).

A total of 224 bacteria were identified at the level of species. Among the identifiable species, *Bacteroides_dorei* ranked the first place in the obesity group and *Faecalibacterium_prausnitzii* ranked the first place in the control group. A total of 27 identifiable species show significant differences between obesity and control groups (Table 3).

**Table 3:** Comparisons of relative abundance of gut bacteria at the level of species in obesity and control groups.

Species	Obesity (Q1, Q3)	Obesity IQR	Control (Q1, Q3)	Control IQR	P-value	FDR
**Species show higher relative abundance in obesity group than in control group**						
Bacteroides_ plebeius	**2**.**414** (**0**.**006**, **10**.**656**)	10.651	0 (0, 0.038)	0.039	0.000	0.000
Parasutterella_ excrementihominis	**0**.**185** (**0**.**015**, **1**.**316**)	1.302	0.010 (0, 0.114)	0.114	0.002	0.020
Parabacteroides_ distasonis	**0**.**522** (**0**.**116**, **0**.**921**)	0.804	0.123 (0.013, 0.328)	0.314	0.002	0.019
Bilophila_ wadsworthia	**0**.**058** (**0**.**004**, **0**.**252**)	0.248	0 (0, 0.077)	0.078	0.005	0.037
Clostridium_ symbiosum	**0**.**074** (**0**.**027**, **0**.**144**)	0.117	0.017 (0, 0.054)	0.054	0.000	0.001
Megamonas_ funiformis	**0** (**0**, **0**.**061**)	0.061	0 (0, 0)	0.000	0.005	0.037
Allisonella_ histaminiformans	**0** (**0**, **0**.**044**)	0.044	0 (0, 0)	0.000	0.000	0.004
Prevotella_ stercorea	**0** (**0**, **0**.**018**)	0.018	0 (0, 0)	0.000	0.004	0.036
Oxalobacter_ formigenes	**0** (**0**, **0**.**003**)	0.003	0 (0, 0)	0.000	0.004	0.034
**Species show higher relative abundance in control than in obesity group**						
Faecalibacterium_ prausnitzii	2.750 (1.134, 5.363)	4.229	**6**.**745** (**2**.**999**, **11**.**682**)	**8**.**683**	0.001	0.008
Bacteroides_ thetaiotaomicron	0.517 (0.084, 1.300)	1.216	**1**.**404** (**0**.**329**, **2**.**710**)	**2**.**381**	0.003	0.030
Blautia_wexlerae	0.139 (0.068, 0.264)	0.196	**1**.**115** (**0**.**380**, **4**.**340**)	**3**.**960**	0.000	0.000
Bacteroides_fragilis	0.049 (0.005, 0.599)	0.595	**0**.**464** (**0**.**103**, **2**.**552**)	**2**.**449**	0.002	0.024
Blautia_luti	0.022 (0.009, 0.063)	0.054	**0**.**292** (**0**.**068**, **0**.**765**)	**0**.**697**	0.000	0.000
Anaerostipes_ butyraticus	0.045 (0.016, 0.193)	0.177	**0**.**234** (**0**.**089**, **1**.**340**)	**1**.**251**	0.000	0.004
Streptococcus_ salivarius	0.050 (0.011, 0.167)	0.156	**0**.**128** (**0**.**047**, **0**.**285**)	**0**.**238**	0.002	0.023
Bacteroides_ ovatus	0.007 (0, 0.039)	0.039	**0**.**086** (**0**.**005**, **1**.**806**)	**1**.**802**	0.000	0.008
Clostridium_ leptum	0 (0, 0.004)	0.004	**0**.**012** (**0**, **0**.**069**)	**0**.**070**	0.000	0.004
Erysipelatoclostridium_ ramosum	0 (0, 0)	0.000	**0**.**005** (**0**, **0**.**117**)	**0**.**118**	0.000	0.000
Lactonifactor_ longoviformis	0 (0, 0.004)	0.004	**0**.**004** (**0**, **0**.**023**)	**0**.**023**	0.000	0.007
Solobacterium_ moorei	0 (0, 0)	0.000	**0**.**003** (**0**, **0**.**021**)	**0**.**021**	0.000	0.000
Akkermansia_ muciniphila	0 (0, 0)	0.000	**0** (**0**, **0**.**040**)	**0**.**040**	0.006	0.044
Enterococcus_ cecorum	0 (0, 0)	0.000	**0** (**0**, **0**.**008**)	**0**.**008**	0.000	0.006
Clostridium_ sporosphaeroides	0 (0, 0)	0.000	**0** (**0**, **0**.**006**)	**0**.**007**	0.001	0.013
Anaerostipes_ caccae	0 (0, 0)	0.000	**0** (**0**, **0**.**004**)	**0**.**004**	0.003	0.025
Streptococcus_ constellatus	0 (0, 0)	0.000	**0** (**0**, **0**.**003**)	**0**.**004**	0.000	0.002
Eubacterium_ sulci	0 (0, 0)	0.000	**0** (**0**, **0**.**003**)	**0**.**004**	0.000	0.003
Gordonibacter_ pamelaeae	0(0, 0)	0.000	0(0, 0)	0	0.001	0.013
Alloprevotella_ rava	0(0, 0)	0.000	0(0, 0)	0	0.003	0.030
**Unclassified**						
Unclassified	**14**.**543** (**10**.**487**, **20**.**586**)	10.099	22.158 (13.915, 31.185)	17.270	0.002	0.019

Data are present as median (quartile Q1, Q3) and interquartile range (IQR = Q3–Q1).

Nine species showed higher relative abundance in obesity group than in control group. These species include *Bacteroides_plebeius*, *Parasutterella_excrementihominis*, *Parabacteroides_distasonis*, *Bilophila_wadsworthia*, *Clostridium_symbiosum*, *Megamonas_funiformis*, *Allisonella_histaminiformans*, *Prevotella_stercorea*, and *Oxalobacter_formigenes*.

Eighteen species showed higher relative abundance in control group than in obesity group. These species include *Faecalibacterium_ prausnitzii*, *Bacteroides_ thetaiotaomicron*, *Blautia_wexlerae*, *Bacteroides_fragilis*, *Blautia_luti*, *Anaerostipes_butyraticus*, *Streptococcus_salivarius*, *Bacteroides_ovatus*, *Clostridium_ leptum*, *Erysipelatoclostridium_ramosum*, *Lactonifactor_longoviformis*, *Solobacterium_moorei*, *Akkermansia_muciniphila*, *Enterococcus_cecorum*, *Clostridium_sporosphaeroides*, *Anaerostipes_caccae*, *Streptococcus_constellatus*, and *Eubacterium_sulci*.

*Gordonibacter_pamelaeae* and *Alloprevotella_rava* showed significant differences between obesity and control groups but both of them have very low relative abundance in obesity and control groups. The overall of remaining unclassified species have a higher relative abundance in control group.

The relative abundance of the remaining194 identifiable species did not show significant differences between the obesity and normal groups (Supplementary Table 1).

## Discussion

In the past decades, studies for microbiome have revealed the critical roles of bacteria in human health and diseases^12^. Sequencing-based analysis for intestinal microbiome is the most advanced methodology used in study for the relationship between microbiome and human health and diseases. With the effort of many scientists, many analysis tools have been established and open to science community to use, the knowledge accumulated in this flied have been explosively increasing^13,14^. With the increase of quality control, the data published by different group may provide more consistent findings^15^. Accordingly, investigations for the correlation between the microbiome and specific disease are greatly interested topics by physicians and scientists. We are greatly motivated to study the relationship between intestinal flora and diseases in children.

Recent experimental studies have reported that there were differences in intestinal flora between obese and healthy animals^16^ as well as between obese mothers and their babies and healthy controls^17^. In the current study, we used second-generation sequencing platform, Illumina Miseq, 16SrDNA high-throughput sequencing technology to characterize qualified faecal DNA from participants. Based on the results of the OTU analysis for the valid 16SrDNA sequences (97% sequence similarity), significant differences of OUT and relative abundance were found between the obesity and the control groups. Our results were consistent with the previously published data and support the notion that intestinal flora may be involved in the development obesity.

The results for α and β diversity analyses indicated that the composition of gut bacteria was significant different between the obesity and control group. While the distinct patterns of diversity were observed between the obesity and control groups, some outliers in the control group were present. Based on these results, we speculated that the complexities in intestinal flora may impacted by numerous factors, such as geographical, ethnic, dietary, lifestyle, DNA degradation^18,19^. The current study was designed with a focus to determine whether there is significant difference of microflora between the obesity and healthy control, we could not analyze the additional factors which may contribute to the formation of different microflora in obesity and control subjects. Because of remarkable of bacterial composition, we are very interested in investigating whether geographical location, ethnic group, dietary patterns, physical activity, hygiene are involved the development of obesity by influencing the formation of different gut microflora.

One interesting finding from this study is that intestinal flora structure has no difference between subjects with BMI ≥ 25 and subjects with BMI < 25. This finding is different from previous finding that there is a significant positive correlation between the levels of proteobacteria and anaerobic Gram-positive bacteria and BMI levels in children^6,20^. We speculate such discrepancy may be due to the different methodology. The previous studies were performed using real-time PCR with focuses on certain species, the diversity of these bacteria were as not analyzed with a data pool of entire gut microbiome. In contrast, the current sequencing techniques provide a data pool to cover the entire microbiome in the gut. Because this sequencing method preserve the integrity of whole microbiome and calculate the amount of different species according to the number of matched sequences to a specific species, the measurement of diversity and relative abundance of the species provide a new algorithm to compare the obesity and control subjects. The advanced Illumina Miseq. 16S rDNA high-throughput sequencing technology can detect structural changes in the intestinal flora of obese school-age children more comprehensively and accurately than other methods. In the meantime, it also has some disadvantages, including relatively expensive cost, higher requirement for DNA quality, and specialized technical staff and hardware implementation. Therefore, each research group should choose proper method based on their own situation^21,22^.

Finally, we analyzed the relative abundance of gut bacteria at multiple levels of classifications. All of the analyses for the differences at the levels of the phylum and species supported the notion that there are distinct pattern of bacterial abundance between obesity and controls groups. Our results were consistent with previous findings. In a Belgian study, the relative proportion of intestinal *Firmicutes* increased in childhood obesity when compared with that of *Bacteroides*^21^. In addition, in a Swedish study, the concentration of Gram-negative *Enterobacteriaceae* in obese/overweight children was significantly higher than children in the control group^22^. In a study of obese Kazak school-age female children in Xinjiang, China, the number of Bacteroidetes was decreased, and the proportion of Firmicutes/Bacteroidetes was increased^23^. In summary, the intestinal flora of obese children may be related to region, race, gender, diet, and experimental conditions. Multi-center research could be carried out when possible to eliminate confounding factors.

Of note, this study has the following limitations. First, the difference of gut bacteria between obesity and control were found on the basis of the relative abundance. However, as discussed in the recent publication by Weiss S *et al*.^24^, more bacterial species may be represented by more number of sequencing rather than the true biological difference. Therefore, a robust process should be adopted to normalizing NGS data to control the bias derived from the differential sequencing efficiency. During our analysis, we excluded sample with low number of sequences (read depth) sample with very small size library, but this process reduced the total sample numbers for the subsequent analyses. Further studies with large sample size should maintain decent analysis power after data normalization. Second, we found the differences of gut bacteria between obesity and control groups at different levels. While these differences were mainly consistent with previous studies, for example, *Akkermansia_muciniphila* and *Faecalibacterium_ prausnitzii* show higher relative abundance in control group than in obesity group, the relative abundance of *Bacteroidetes* was higher in obesity group and the relative abundance of *Firmicutes* was higher in control group. These variation may be due to the difference of methods, or study subjects. More studies with different populations and large scale meta-analysis may allow us to understand the factors that may cause the variations of the results in different studies^25^.

## Conclusion

The study for intestinal flora has become the new frontier in understanding the development of obesity. The current study explored the relationship between intestinal flora and obesity in school-age children. Our results indicated that intestinal flora in obese children showed lower diversity than normal controls. In addition, the relative abundance of intestinal flora showed distinct pattern at multiple levels of classifications between obesity and control groups. Investigation of each of species with significant difference between obese and normal children may provide important information to uncover the roles of these specific bacteria in the development of obesity and find new strategy to prevent and treat obesity through intervening the intestinal flora.

## Methods

### Study design and subjects

This study was reviewed and approved by institutional review board (IRB) of West China Second University Hospital, all methods were performed in accordance with the relevant guidelines and regulations set by ethnic committee, and written informed consent was obtained from the guardians of the subjects.

The inclusion criteria were as follows: obese school-age children (meeting the diagnostic criteria of WHO/NCHS standards^26,27^) who were seen at the Children Health Care Out-patient Clinic of West China Second University Hospital of Sichuan University were included in the obesity group. Healthy children were selected and defined as the control group. The exclusion criteria were as follows: (1) having used antibiotics or probiotics within the past 4 weeks; (2) having gastrointestinal diseases (e.g., diarrhoea) within the past 4 weeks; and (3) not agreeing to participate in this study.

Basic participant data, including age, gender and family history of obesity, heights (m) and body weights (kg) were recorded.

### Fecal sample processing and 16S rDNA High-throughput Sequencing

Using sterile fecal boxes, fresh feces (5 g) expelled within 2 h were collected from obesity and control children. Fecal samples were quickly placed in ultra-low temperature freezer (−80 °C) for further processing and testing.

Fecal DNA was extracted using QIAamp DNA Stool Mini Procedure Fecal Extraction DNA Kit (QIAGEN, Germany). DNA concentration and the ratios of absorbance at 260 nm vs 280 nm (A260 nm/280 nm) were determined by UV spectrophotometer. The integrity of the DNA fragments was detected by 1% agarose gel electrophoresis (voltage: 150 V, time: 40 min), and samples were stored in −20°C freezer for further analysis.

Qualified DNA samples were sent to BGI Co. overnight under refrigeration for 16S rDNA V3-V4 hypervariable region PCR amplification, library construction, and Illumina Miseq. 2000 16S rDNA high-throughput sequencing. The forward (341 bp) and reverse (806 bp) PCR primers were 5′ACTCCTACGGGAGGCAGCAG 3′ and 5′GGACTACHVGGGTWTCTAAT 3′, respectively. The final reaction volume of 50 μL was composed of 30 ng DNA, 2 μL of both primers, and 25 μL PCR mixture, and ddH2O added until the 50 μL volume was reached. The amplification cycle consisted of pre-denaturation at 98 °C for 3 min, 30 cycles of denaturation (98 °C for 45 s), annealing (55 °C for 45 s), extension (72 °C for 45 s), secondary extension at 72 °C for 10 min, and final extension at 72 °C for 7 min. The construction of the high-throughput sequencing library and execution of the Illumina Miseq. 2000 16SrDNA high-throughput sequencing followed the following process: We prepared PCR products for sequencing and built the PCR library using the Illumina Stool DNA Library Kit and Tru Seq DNA LT Sample Prep Kit according to the kits’ instructions. Double-ended sequencing was performed on the PCR products using the Illumina Miseq platform.

### Bioinformatics Analysis

The raw data for the 16S rDNA high-throughput sequencing were firstly subjected to the quality analysis using the designated software, ensuring only data meeting the quality criteria for sequencing depth, coverage and uniformity were used for the further analysis for classification, abundance and diversity analyses (BGI Tech Co.). The analysis software/methods include Heatmap, Principal coordinates analysis (PCoA), Claster, and Metastates. To reduce the bias caused by differential sequencing efficiency, samples that have low read depth and the samples below the 15^th^ percentile of library size in the subsequent analyses were excluded^24^.

### Statistical analysis

The data were analyzed and compared using the SPSS package. The normally distributed measurement data were expressed as median (interquartile range). The paired *t-*test was employed for comparison of mean numbers of randomly designed samples before and after the treatment. Differences in microbial community abundance between the obesity group and the control group were measured using the rank sum test and wilcox. test, and the significance of these differences was assessed using FDR (False discovery rate). *P* ≤ 0.05 indicated statistical significance.

## Electronic supplementary material

Supplementary Dataset 1

## Data Availability

Materials, data and associated protocols are promptly available to readers without undue qualifications in material transfer agreements.
