# Supplementary material for: A study of the correlation between obesity and intestinal flora in school-age children
Source: Sci Rep. 2018 Sep 28;8:14511. doi: 10.1038/s41598-018-32730-6 (PMC6162261; doi:10.1038/s41598-018-32730-6)
Supplement: Supplementary file 1 — Supplementary Dataset 1 [file 41598_2018_32730_MOESM1_ESM.pdf]

A study of the correlation between obesity and intestinal flora in school-age children

Xiaolin Gao[1,5], MD, Ruizhen Jia [2], MM, Liang Xie [3], PhD, Linghan Kuang [4], MM, Ling Feng [1], MM, Chaomin Wan[1], MD, PhD

Affiliations:

[1] Department of Paediatrics, West China University Second Hospital, Sichuan University (Chengdu, Sichuan, China, 610041).

[2] Open Laboratory, West China Institute for Women's and Children's Health (Chengdu, Sichuan, China, 610041).

[3] Pulmonary Vascular Remodelling Research Unit Laboratory, West China Institute for Women's and Children's Health (Chengdu, Sichuan, China, 610041).

[4] Department of Laboratory Medicine, Sichuan University West China Second Hospital (Chengdu, Sichuan, China, 610041).

[5] Key Laboratory of Birth Defects and Related Diseases of Women and Children (Sichuan University), Ministry of Education

Corresponding author:

Chaomin Wan [1]MD, PhD

Professor, Department of Paediatrics

Western Women's and Children's Research Institute

Number 20, 3rd Section, People's South Road

Chengdu, Sichuan Province, China, 610041

Email: wanchaomin@scu.edu.cn

TEL: +86 2885503185

Fax: +86 2885503799.

Authors' Email:

Xiaolin Gao: 23132875@qq.com

Ruizhen Jia: 1002113878@qq.com

Liang Xie: 1154792673@qq.com

Linghan Kuang: hanhan840106@163.com

Ling Feng: 348030966@qq.com

Chaomin Wan: wanchaomin@scu.edu.cn

Supplementary information

Supplementary Figure 1. Representative graph shows the rarefaction of sample (Shannon).The similar rarefaction measures for other indexes (observed species index, ACE index, and Simpson index) were performed in the same manner.

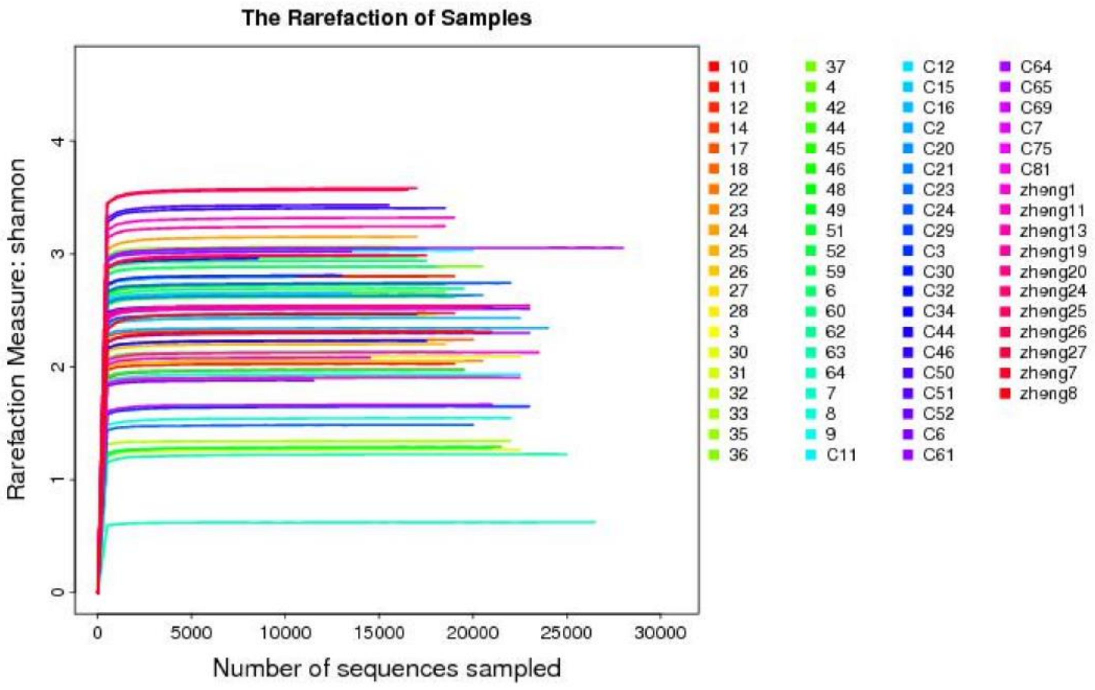

Supplementary Table 1. Comparisons of relative abundance of gut bacteria at the level of species in obesity and control groups. Data are present as median (quartile Q1, Q3) and interquartile range (IQR)

Species	Obesity	Obesity IQR	Control	Control IQR	P-value	FDR
Coprobacillus_cateniformis	0 (0,0)	0.000	0 (0,0.00407275)	0.004	0.011	0.077
Staphylococcus_capitis	0 (0,0)	0.000	0 (0,0)	0.000	0.012	0.085
Blautia_hansenii	0 (0,0)	0.000	0 (0,0)	0.000	0.013	0.088
Parvimonas_micra	0 (0,0)	0.000	0 (0,0.00272625)	0.003	0.013	0.088
Bifidobacterium_breve	0 (0,0)	0.000	0 (0,0.00867875)	0.009	0.014	0.092
Terrisporobacter_glycolicus	0 (0,0)	0.000	0 (0,0.004059)	0.004	0.015	0.096
Dialister_succinatiphilus	0 (0,0)	0.000	0 (0,0)	0.000	0.016	0.097
Anaerotruncus_colihominis	0 (0,0)	0.000	0 (0,0.0114675)	0.011	0.018	0.104
Lactobacillus_rogosae	0.0533995 (0.0059865, 0.26305025)	0.257	0.0122135 (0,0.0575285)	0.058	0.018	0.106
Oribacterium_sinus	0 (0,0)	0.000	0 (0,0)	0.000	0.023	0.129
Clostridium_colinum	0 (0,0)	0.000	0 (0,0)	0.000	0.024	0.130
Bacteroides_dorei	7.3311575 (1.6936,23.91409225)	22.220	2.039316 (0.529311,8.78872)	8.259	0.026	0.132
Eubacterium_siraeum	0 (0,0)	0.000	0 (0,0.01680775)	0.017	0.025	0.132
Ruminococcus_lactaris	0 (0,0)	0.000	0 (0,0.004556)	0.005	0.025	0.132
Dialister_invisus	0 (0,0.01811875)	0.018	0.026985 (0,0.509202)	0.509	0.027	0.132
Streptococcus_mutans	0 (0,0)	0.000	0 (0,0)	0.000	0.027	0.132
Ruminococcus_torques	0.19293 (0.0515635, 0.4552475)	0.404	0.291623 (0.1101765,0.986532)	0.876	0.034	0.164
Clostridium_spiroforme	0 (0,0)	0.000	0 (0,0)	0.000	0.035	0.166
Brevundimonas_vesicularis	0 (0,0)	0.000	0 (0,0)	0.000	0.043	0.177

Lactobacillus_gasseri	0 (0,0)	0.000	0 (0,0)	0.000	0.043	0.177
Mitsuokella_multacida	0 (0,0)	0.000	0 (0,0)	0.000	0.043	0.177
Parabacteroides_johnsonii	0 (0,0)	0.000	0 (0,0)	0.000	0.042	0.177
Phascolarctobacterium_faecium	0.6267315 (0.05078375,1.92924375)	1.878	0.0996425 (0.00484175,0.7122665)	0.707	0.040	0.177
Roseburia_hominis	0 (0,0)	0.000	0 (0,0)	0.000	0.041	0.177
Rothia_mucilaginosa	0 (0,0)	0.000	0 (0,0)	0.000	0.039	0.177
Bifidobacterium_bifidum	0 (0,0)	0.000	0 (0,0)	0.000	0.047	0.188
Bacteroides_caccae	0.0264705 (0.00104,0.1956335)	0.195	0.121857 (0.00749175,0.56704825)	0.560	0.049	0.192
Blautia_faecis	0.0233625 (0.0043085, 0.04447975)	0.040	0.0404905 (0.00783675,0.0910785)	0.083	0.053	0.204
Atopobium_parvulum	0 (0,0)	0.000	0 (0,0)	0.000	0.056	0.215
Megasphaera_micronuciformis	0 (0,0)	0.000	0 (0,0)	0.000	0.066	0.246
Acinetobacter_johnsonii	0 (0,0)	0.000	0 (0,0)	0.000	0.073	0.253
Acinetobacter_venetianus	0 (0,0)	0.000	0 (0,0)	0.000	0.082	0.253
Alcaligenes_faecalis	0 (0,0)	0.000	0 (0,0)	0.000	0.082	0.253
Alistipes_finegoldii	0 (0,0)	0.000	0 (0,0)	0.000	0.082	0.253
Caulobacter_segnis	0 (0,0)	0.000	0 (0,0)	0.000	0.082	0.253
Clostridium_saccharogumia	0 (0,0)	0.000	0 (0,0)	0.000	0.082	0.253
Corynebacterium_argentoratense	0 (0,0)	0.000	0 (0,0)	0.000	0.082	0.253
Eubacterium_brachy	0 (0,0)	0.000	0 (0,0)	0.000	0.082	0.253
Hungatella_hathewayi	0 (0,0.00750875)	0.008	0.001356 (0,0.01611975)	0.016	0.080	0.253
Lactococcus_l	0 (0,0)	0.000	0 (0,0)	0.000	0.082	0.253

actis						
Lactococcus_raffinolactis	0 (0,0)	0.000	0 (0,0)	0.000	0.082	0.253
Mogibacterium_neglectum	0 (0,0)	0.000	0 (0,0.00341725)	0.003	0.073	0.253
Rhizobium_tropici	0 (0,0)	0.000	0 (0,0)	0.000	0.082	0.253
Sutterella_parvirubra	0 (0,0)	0.000	0 (0,0)	0.000	0.086	0.263
Bacteroides_nordii	0 (0,0.02124475)	0.021	0.006342 (0,0.0572795)	0.057	0.094	0.275
Bifidobacterium_animalis	0 (0,0)	0.000	0 (0,0)	0.000	0.093	0.275
Fusobacterium_nucleatum	0 (0,0)	0.000	0 (0,0)	0.000	0.093	0.275
Actinomyces_odontolyticus	0 (0,0)	0.000	0 (0,0.005011)	0.005	0.098	0.279
Clostridium_perfringens	0 (0,0.00881475)	0.009	0 (0,0)	0.000	0.099	0.279
Parabacteroides_goldsteinii	0 (0,0)	0.000	0 (0,0)	0.000	0.100	0.279
Veillonella_atypica	0 (0,0)	0.000	0 (0,0)	0.000	0.101	0.279
Veillonella_dispar	0.071067 (0.0172175, 0.23985775)	0.223	0.029957 (0.00804125,0.121743)	0.114	0.107	0.293
Acinetobacter_lwoffii	0 (0,0)	0.000	0 (0,0)	0.000	0.160	0.332
Alistipes_putredinis	0 (0,0)	0.000	0 (0,0)	0.000	0.160	0.332
Bacteroides_fluxus	0 (0,0)	0.000	0 (0,0)	0.000	0.162	0.332
Butyricimonas_synergistica	0 (0,0)	0.000	0 (0,0)	0.000	0.160	0.332
Centipeda_periodontii	0 (0,0)	0.000	0 (0,0)	0.000	0.160	0.332
Clostridium_aldenense	0 (0,0)	0.000	0 (0,0)	0.000	0.160	0.332
Clostridium_paraputrificum	0 (0,0)	0.000	0 (0,0)	0.000	0.148	0.332
Collinsella_aerofaciens	0.009539 (0,0.044829)	0.045	0.0043425 (0,0.0198135)	0.020	0.145	0.332
Collinsella_intestinalis	0 (0,0)	0.000	0 (0,0)	0.000	0.140	0.332

Coprococcus_eutactus	0 (0,0)	0.000	0 (0,0.02271325)	0.023	0.146	0.332
Delftia_tsuruhatensis	0 (0,0)	0.000	0 (0,0)	0.000	0.160	0.332
Dorea_longicatena	0.067087 (0.02327275,0.13833725)	0.115	0.0441475 (0.0009465,0.158742)	0.158	0.159	0.332
Enhydrobacter_aerosaccus	0 (0,0)	0.000	0 (0,0)	0.000	0.160	0.332
Flavobacterium_suncheonense	0 (0,0)	0.000	0 (0,0)	0.000	0.160	0.332
Fluviicola_taffensis	0 (0,0)	0.000	0 (0,0)	0.000	0.160	0.332
Gemmiger_formicilis	0.3307995 (0.0599675, 0.665856)	0.606	0.874484 (0.0096125,2.37034125)	2.361	0.151	0.332
Granulicatella_adiacens	0 (0,0.004214)	0.004	0 (0,0.0051985)	0.005	0.151	0.332
Holdemania_filiformis	0 (0,0)	0.000	0 (0,0.0073315)	0.007	0.144	0.332
Lachnoanaerobaculum_umeaense	0 (0,0)	0.000	0 (0,0)	0.000	0.160	0.332
Lactobacillus_mucosae	0 (0,0)	0.000	0 (0,0)	0.000	0.160	0.332
Leuconostoc_lactis	0 (0,0)	0.000	0 (0,0)	0.000	0.161	0.332
Megasphaera_paucivorans	0 (0,0)	0.000	0 (0,0)	0.000	0.160	0.332
Methylobacterium_jeotgali	0 (0,0)	0.000	0 (0,0)	0.000	0.160	0.332
Paracoccus_caeni	0 (0,0)	0.000	0 (0,0)	0.000	0.160	0.332
Propionibacterium_acnes	0 (0,0)	0.000	0 (0,0)	0.000	0.160	0.332
Rothia_dentocariosa	0 (0,0)	0.000	0 (0,0)	0.000	0.160	0.332
Ruminococcus_albus	0 (0,0)	0.000	0 (0,0)	0.000	0.160	0.332
Sphaerotilus_natans	0 (0,0)	0.000	0 (0,0)	0.000	0.160	0.332
Sutterella_wadsworthensis	0 (0,0)	0.000	0 (0,0)	0.000	0.167	0.339
Ruminococcus_faecis	0.00749 (0,0.022555	0.023	0.0096415 (0.0039785,0.0	0.044	0.170	0.341

	)		4815275)			
Cloacibacillus_evryensis	0 (0,0)	0.000	0 (0,0)	0.000	0.180	0.358
Fusobacterium_varium	0 (0,0)	0.000	0 (0,0)	0.000	0.184	0.362
Coprococcus_comes	0.090711 (0.008028,0.2138515)	0.206	0.0519835 (0.00534525,0.14250075)	0.137	0.214	0.418
Anaerofustis_stercorihominis	0 (0,0)	0.000	0 (0,0)	0.000	0.225	0.432
Megasphaera_elsdenii	0 (0,0)	0.000	0 (0,0)	0.000	0.225	0.432
Acidaminococcus_fermentans	0 (0,0)	0.000	0 (0,0)	0.000	0.231	0.435
Prevotella_copri	0.0100535 (0,18.21423375)	18.214	0.005152 (0,0.1351025)	0.135	0.232	0.435
Turicibacter_sanguinis	0 (0,0)	0.000	0 (0,0.005164)	0.005	0.231	0.435
Abiotrophia_defectiva	0 (0,0)	0.000	0 (0,0)	0.000	0.259	0.447
Anaerofilum_pentosovorans	0 (0,0)	0.000	0 (0,0)	0.000	0.328	0.447
Bacillus_coagulans	0 (0,0)	0.000	0 (0,0)	0.000	0.328	0.447
Bacillus_thermoamylovorans	0 (0,0)	0.000	0 (0,0)	0.000	0.328	0.447
Bacteroides_barnesiae	0 (0,0)	0.000	0 (0,0)	0.000	0.328	0.447
Bacteroides_coprophilus	0 (0,0)	0.000	0 (0,0)	0.000	0.327	0.447
Bacteroides_intestinalis	0 (0,0)	0.000	0 (0,0)	0.000	0.256	0.447
Bacteroides_massiliensis	0 (0,0.004328)	0.004	0 (0,0)	0.000	0.252	0.447
Bacteroides_sartorii	0 (0,0)	0.000	0 (0,0)	0.000	0.328	0.447
Bacteroides_stercoris	0.3065525 (0.0138375,2.110356)	2.097	0.0904885 (0.008692,0.9058375)	0.897	0.273	0.447
Bifidobacterium_adolescentis	0 (0,0)	0.000	0 (0,0)	0.000	0.328	0.447

Blautia_obeum	0 (0,0.00459925)	0.005	0 (0,0.0056475)	0.006	0.285	0.447
Campylobacter_hominis	0 (0,0)	0.000	0 (0,0)	0.000	0.320	0.447
Campylobacter_jejuni	0 (0,0)	0.000	0 (0,0)	0.000	0.328	0.447
Catenibacterium_mitsuokai	0 (0,0)	0.000	0 (0,0)	0.000	0.320	0.447
Cellulosilyticum_ruminicola	0 (0,0)	0.000	0 (0,0)	0.000	0.328	0.447
Chryseobacterium_hominis	0 (0,0)	0.000	0 (0,0)	0.000	0.328	0.447
Cloacibacterium_normanense	0 (0,0)	0.000	0 (0,0)	0.000	0.328	0.447
Clostridium_bartlettii	0.0099195 (0.00399525,0.05182775)	0.048	0.0181015 (0.0059,0.049472)	0.044	0.281	0.447
Clostridium_lactatifermentans	0.0048945 (0,0.01425525)	0.014	0 (0,0.013063)	0.013	0.282	0.447
Clostridium_scindens	0 (0,0.004986)	0.005	0 (0,0.01291725)	0.013	0.302	0.447
Daeguia_caeni	0 (0,0)	0.000	0 (0,0)	0.000	0.328	0.447
Eubacterium_callanderi	0 (0,0)	0.000	0 (0,0)	0.000	0.266	0.447
Gemella_sanguinis	0 (0,0.0046605)	0.005	0 (0,0.0080245)	0.008	0.280	0.447
Helicobacter_equorum	0 (0,0)	0.000	0 (0,0)	0.000	0.328	0.447
Lactobacillus_paracasei	0 (0,0)	0.000	0 (0,0)	0.000	0.328	0.447
Lactobacillus_ruminis	0 (0,0)	0.000	0 (0,0)	0.000	0.299	0.447
Megamonas_rupellensis	0 (0,0)	0.000	0 (0,0)	0.000	0.328	0.447
Methylobacterium_komagatae	0 (0,0)	0.000	0 (0,0)	0.000	0.328	0.447
Negativicoccus_succinicivorans	0 (0,0)	0.000	0 (0,0)	0.000	0.328	0.447
Paraprevotella_xylaniphila	0 (0,0)	0.000	0 (0,0)	0.000	0.328	0.447

Phocaeicola_abscessus	0 (0,0)	0.000	0 (0,0)	0.000	0.328	0.447
Plesiomonas_shigelloides	0 (0,0)	0.000	0 (0,0)	0.000	0.328	0.447
Pseudoxanthomonas_taiwanensis	0 (0,0)	0.000	0 (0,0)	0.000	0.328	0.447
Ralstonia_insidiosa	0 (0,0)	0.000	0 (0,0)	0.000	0.328	0.447
Ralstonia_pickettii	0 (0,0)	0.000	0 (0,0)	0.000	0.328	0.447
Raoultella_ornithinolytica	0 (0,0)	0.000	0 (0,0)	0.000	0.245	0.447
Rheinheimera_texasensis	0 (0,0)	0.000	0 (0,0)	0.000	0.328	0.447
Ruminococcus_callidus	0 (0,0.09840975)	0.098	0.0104055 (0,0.13720775)	0.137	0.321	0.447
Shinella_kummerowiae	0 (0,0)	0.000	0 (0,0)	0.000	0.299	0.447
Sphingomonas_yunnanensis	0 (0,0)	0.000	0 (0,0)	0.000	0.328	0.447
Tepidimonas_fonticaldi	0 (0,0)	0.000	0 (0,0)	0.000	0.328	0.447
Thermicanus_aegyptius	0 (0,0)	0.000	0 (0,0)	0.000	0.328	0.447
Truepera_radiovictrix	0 (0,0)	0.000	0 (0,0)	0.000	0.328	0.447
Uruburuella_suis	0 (0,0)	0.000	0 (0,0)	0.000	0.328	0.447
Klebsiella_pneumoniae	0 (0,0.00522675)	0.005	0.0018175 (0,0.01463575)	0.015	0.333	0.448
Parasutterella_secunda	0 (0,0)	0.000	0 (0,0)	0.000	0.331	0.448
Sutterella_stercoricanis	0 (0,0)	0.000	0 (0,0)	0.000	0.335	0.449
Eubacterium_coprostanoligenes	0.047684 (0.004136,0.5270615)	0.523	0.0377615 (0,0.2299035)	0.230	0.342	0.456
Butyricicoccus_pullicaecorum	0.0233335 (0.00462775,0.10223775)	0.098	0.0343085 (0.013933,0.09428925)	0.080	0.353	0.468
Clostridium_viride	0 (0,0.004319)	0.004	0 (0,0.0085825)	0.009	0.355	0.468

Actinomyces_graevenitzii	0 (0,0)	0.000	0 (0,0)	0.000	0.396	0.512
Paraprevotella_clara	0 (0,0)	0.000	0 (0,0)	0.000	0.392	0.512
Ruminococcus_bromii	0.4668695 (0.08067775,1.68742625)	1.607	0.283268 (0.01821225,1.41034675)	1.392	0.396	0.512
Alistipes_onderdonkii	0.050782 (0.005135,0.26589925)	0.261	0.0318795 (0,0.4652535)	0.465	0.406	0.520
Slackia_piriformis	0 (0,0)	0.000	0 (0,0)	0.000	0.406	0.520
Bacteroides_finegoldii	0 (0,0)	0.000	0 (0,0)	0.000	0.417	0.527
Granulicatella_elegans	0 (0,0)	0.000	0 (0,0)	0.000	0.417	0.527
Clostridium_asparagiforme	0 (0,0)	0.000	0 (0,0)	0.000	0.443	0.557
Dialister_pneumosintes	0 (0,0)	0.000	0 (0,0)	0.000	0.453	0.566
Parabacteroides_merdae	0.1401575 (0.005724,0.55318125)	0.547	0.117282 (0,0.39638)	0.396	0.459	0.570
Coprococcus_catus	0 (0,0.0124235)	0.012	0 (0,0.0148015)	0.015	0.463	0.573
Bacteroides_salyersiae	0 (0,0.02013425)	0.020	0.0042725 (0,0.012037)	0.012	0.466	0.573
Sarcina_ventriculi	0 (0,0)	0.000	0 (0,0)	0.000	0.482	0.589
Roseburia_inulinivorans	0.208197 (0.05144575,0.938633)	0.887	0.1366435 (0.018166,1.0091595)	0.991	0.489	0.594
Desulfovibrio_piger	0 (0,0)	0.000	0 (0,0)	0.000	0.505	0.610
Alistipes_indistinctus	0.001961 (0,0.05924525)	0.059	0 (0,0.032919)	0.033	0.533	0.642
Lactobacillus_oris	0 (0,0)	0.000	0 (0,0)	0.000	0.551	0.656
Roseburia_faecis	0 (0,0)	0.000	0 (0,0)	0.000	0.549	0.656
Clostridium_citroniae	0 (0,0)	0.000	0 (0,0)	0.000	0.568	0.659
Kocuria_palustris	0 (0,0)	0.000	0 (0,0)	0.000	0.568	0.659

Odoribacter_laneus	0 (0,0)	0.000	0 (0,0)	0.000	0.568	0.659
Parabacteroides_gordonii	0 (0,0)	0.000	0 (0,0)	0.000	0.568	0.659
Prevotella_timonensis	0 (0,0)	0.000	0 (0,0)	0.000	0.568	0.659
Bacteroides_xylanisolvens	1.0439275 (0.16902025,3.12830475)	2.959	0.9058265 (0.1975215,2.30964025)	2.112	0.578	0.667
Oscillibacter_valericigenes	0.094937 (0.02261125,0.18905375)	0.166	0.078005 (0.0135505,0.285895)	0.272	0.587	0.671
Ruminococcus_flavefaciens	0 (0,0)	0.000	0 (0,0)	0.000	0.585	0.671
Escherichia_fergusonii	0.070175 (0.013279,0.2873375)	0.274	0.12032 (0,0.27019575)	0.270	0.605	0.687
Victivallis_vadensis	0 (0,0)	0.000	0 (0,0)	0.000	0.612	0.692
Alistipes_shahii	0.015845 (0,0.20226075)	0.202	0.007859 (0,0.257981)	0.258	0.656	0.738
Bacteroides_rodentium	0 (0,0)	0.000	0 (0,0)	0.000	0.669	0.749
Prevotella_disiens	0 (0,0)	0.000	0 (0,0)	0.000	0.684	0.762
Flavonifractor_plautii	0.035952 (0.00894275,0.1055155)	0.097	0.0347135 (0.0065955,0.080181)	0.074	0.687	0.762
Bacteroides_cellulosilyticus	0.004209 (0,0.04126725)	0.041	0 (0,0.03470675)	0.035	0.712	0.785
Lactobacillus_salivarius	0 (0,0)	0.000	0 (0,0)	0.000	0.715	0.785
Pyramidobacter_piscolens	0 (0,0)	0.000	0 (0,0)	0.000	0.756	0.826
Bacteroides_coprocola	0 (0,0.022167)	0.022	0 (0,0.07804625)	0.078	0.778	0.846
Barnesiella_intestinihominis	0 (0,0.1068355)	0.107	0 (0,0.06300675)	0.063	0.820	0.885
Butyricimonas_virosa	0 (0,0.01861675)	0.019	0 (0,0.0107795)	0.011	0.822	0.885
Haemophilus_parainfluenzae	0.0265145 (0,0.15473025)	0.155	0.0250525 (0.00491975,0.13211375)	0.127	0.839	0.898

Acinetobacter_beijerinckii	0 (0,0)	0.000	0 (0,0)	0.000	1.000	1.000
Bacteroides_clarus	0 (0,0)	0.000	0 (0,0)	0.000	0.974	1.000
Bacteroides_eggerthii	0 (0,0)	0.000	0 (0,0)	0.000	0.987	1.000
Bacteroides_uniformis	0.922804 (0.08522875,3.575247)	3.490	0.8454785 (0.10672025,3.57849425)	3.472	0.941	1.000
Clostridium_methylpentosum	0 (0,0)	0.000	0 (0,0)	0.000	1.000	1.000
Collinsella_tanakaei	0 (0,0)	0.000	0 (0,0)	0.000	1.000	1.000
Eggerthella_lenta	0 (0,0.00506)	0.005	0 (0,0.00606725)	0.006	0.958	1.000
Finegoldia_magna	0 (0,0)	0.000	0 (0,0)	0.000	1.000	1.000
Lactobacillus_sanfranciscensis	0 (0,0)	0.000	0 (0,0)	0.000	1.000	1.000
Peptococcus_niger	0 (0,0)	0.000	0 (0,0)	0.000	1.000	1.000
Peptoniphilus_lacrimalis	0 (0,0)	0.000	0 (0,0)	0.000	1.000	1.000
Porphyromonas_bennonis	0 (0,0)	0.000	0 (0,0)	0.000	0.974	1.000
Pseudoflavonifractor_capillosus	0 (0,0)	0.000	0 (0,0)	0.000	1.000	1.000
Veillonella_rogosae	0 (0,0)	0.000	0 (0,0)	0.000	1.000	1.000
